# On the Uses of the Binder in Parturient Women

**Published:** 1875-09

**Authors:** P. H. Clarke

**Affiliations:** Mason City, West Va.


### Southern Medical Record.

VOL. V.—SEPTEMBER, 1875.—No. 9.

### Original Communications.

### ON THE USES OF THE BINDER IN PARTURIENT WOMEN.

BY P. H. CLARKE, M. D., MASON CITY, WEST VA.

*Read before the Meigs County, Ohio, Medical Association at Syracuse, Ohio, at its May meeting,* 1875, *and requested to be published in the Southern Medical Record.*

The use of the binder in parturient females, in one way and another, has continued from some very remote point in the history of midwifery up to the present. We are unable to fix definitely and precisely the time of its original adoption into the practice, for the modes of recording clinical experience, in earlier times, were so imperfect; besides, there existed such a thorough prejudice to engaging in this branch of professional work, as effectually to forbid the espousal of its grave responsibilities and interests by that better class of talented men, into whose hands this important work has fallen in these latter and brighter days. The bandage has been used in parturients for two distinct purposes, namely: (1) to effect pressure, during the progress of labor, upon the contents of the womb, through the walls of the abdomen. (2) To meet certain supposed indications after the completion of the third stage of labor, the safe delivery of the child and placenta, with its appendages. In a few years' work, as a practioner of medicine, I have met with a very large proportional amount of experience in the branch of obstetrics. And, whilst the individual experience of no one, though covering a great number of years, can enable him to speak "ex cathedra" on all points relating to this extensive branch of practice, it will, nevertheless, not fail to improve him and benefit others a vast deal in many ways. From my own experience, I have surely derived a positive corroboration of the views of other observers upon certain important points, whilst some deductions of my own, twenty years hence, in the light of extended experience and observation, I may find it necessary to reject as worthless. It is a fact well known and admitted that the vast majority of cases of childbirth will terminate favorably to both mother and offspring, if left alone to the unaided powers of nature. And whilst some labors are very tedious, and frequently terminate rapidly by artificial means, it is by no means certain that the same labor would not have terminated properly had it been left alone. And, I am ready to admit, so it may have been in some cases of my own which were progressing very slowly, and *tormentingly*, until I applied pressure to the abdomen, by means of the binder, and the labors were at once completed, and always without one solitary untoward symptom presenting itself. In the first case in which I witnessed the result of pressure applied ante-partum everything in the case seemed normal, excepting the extreme tardiness of the progressive movements of the child. The os uteri was fully dilated, and the maternal passages ample, and the tissues fully softened and distensible. I had been endeavoring to stimulate the uterus to more forcible contraction, as my habit is, by manipulating the walls of the uterus through the parietes abdominis. Finally I directed an old German female attendant to perform this movement whilst I observed the effects of it digitally. She proceeded to execute my behests in a manner somewhat alarmingly vigorous, making *forcible pressure,* as she said she had frequently done before, on other and similar occasions. The child was expelled before I could call upon her to desist, and in a manner fully to satisfy me that it was the direct effect of the pressure. Since that time I have resorted to methodical pressure in several instances in very delicate subjects in whom labor was progressing very tardily, and always with the promptest and most satisfactory results. I do not think of vindicating the use of the binder during labor in all cases, but speak of it in order to commend its use in certain selected cases in which you would prefer not to administer ergot, (and even in some cases where this agent has been used, and you would otherwise have to resort to forceps,) and in which you desire the speediest results consistent with the safety of the patient. At a meeting of the Obstetric Society, at Edinburg, Feb 12, 1873, the subject of mechanical aids to labor came up, and pressure ranked most prominently among the means suggested. W. Ash Smith was satisfied that he had saved his patients many hours of suffering, and on more than one occasion had obviated the use of the forceps by the timely employment of pressure. He said use the hand, for convenience sake, and, when it tires, use the binder. He believed the girdles which were placed around parturient women, by the ancient Britons, were originally used to effect pressure, but they had, in course of time, degenerated into charms and lost their usefulness. Prof. Simpson still used, with success, a binder designed by Dr. Protheroe Smith, by which constant and increasing pressure was kept up. Dr. McRae said, a common practice of his was to bind, giving the binder one twist at the back, as each pain came on. Dr. Burn used pressure in tedious labors and pendulous bellies; whilst Dr. Young cordially advocated pressure with the left hand over the fundus uteri during each expulsive pain, by which means he found tedious labors, especially, greatly accelerated, and the patient very considerably relieved.

It is a fact known to students of history that the ancients so adjusted their clothing as to throw the onus of supporting its weight upon the shoulders instead of the waist, where modern usage has placed it, thus exhibiting a shade of sound philosophy which might, with benefit, be patterned after by the present generation. This reform, however, is not likely to take place very soon, as it has not existed for a number of years in the past, and the weight of female dress, which is somewhat greater now than it was, even in the days of our grand-mothers, will continue to be suspended from the waist, and its pernicious effects continued. The abdominal, thoracic and lumbo spinal muscles are subjected, through this agency, aided in many instances by tight lacing, to constant and significant pressure, the result of which is, *sometimes degeneration* of the muscular structures, but *always* enfeeblement of them through attenuation of the fibre from absorption, one of the inevitable results of pressure. Thus, when the time arrives when the parturient would essay to give voluntary aid to her expulsive pains through these muscles, they are found inadequate to meet the ends which, under normal conditions, they powerfully subserve. And, in order that the uterine forces, *per se*, which are vastly overtaxed, may be aided, there is no resource left but to supply artificially the tone, vigor and elastic strength which the structures have lost, by a properly adapted binder. In these cases this practice will invariably yield the happiest results, when it is resorted to with the promptitude which the necessities of such a condition imperatively demand. Then, not in the first stage of labor could there be any reason for the application of the binder, for the process of uterine dilatation is marching ahead through the agency of the intrinsic forces of this organ acting upon its contents. But after the os is thoroughly dilated and the presenting part is firmly and surely engaged, the pains designated by the term "dilators" give way to the expulsive pains, and we now witness the phenomena which positively indicate the importance of the use of the ante-partum binder in properly selected cases. All the auxiliary means which are attainable by the earnest parturient to assist the inherent forces of the womb itself are now brought into action.

The abdominal walls are steadied and retracted, co-incidently with the repetition of forced inspiration, through which the lungs are so fully inflated with atmospheric air that a firm point of support is thus gained, against which the diaphragm, acting as a lever of the first kind, finds a fulcrum, and thus is gained a firm and efficient aid to the expulsive efforts of the womb, in women who represent a normal physiological condition. But when the muscles are soft, flabby and attenuated by the various causes of disease, improper modes of dress, &c., then the efforts of the parturient female to subsidize these auxiliary means fail in a great degree to be attended by the success which we witness in strong women, and we are instinctively led to supply the delinquency as well as we can with the means at our command. And nothing will substitute the rigid walls of the abdomen but a well adapted binder, and pads adjusted in accordance with accepted principles of mechanical philosophy.

In turning to the consideration of the binder as a post-partum measure, we are at once met with the fact that it is an appliance which has been used for a long time, by the people, and is recommended by and taught in the most impressive manner by the medical and surgical teachers of the country, generally, who expend a vast deal of time and pains in order to insure the faithful attending to it by all the professional fledglings. It is taught by several distinguished writers and authors of Text, as follows, namely: P. Cazeaux, in his admirable work on midwifery, treats this subject curtly, as follows: "There is a custom much in vogue of surrounding the belly with a moderately tightened bandage, and the women, for the most part, attach the highest importance to this measure as a preservative against the wrinkles and folds that are found after labor, on the skin of the abdomen, as also to prevent the latter from remaining too voluminous. Their desires may be yielded to the more readily, as such a bandage, when moderately drawn, supplies the pressure no longer afforded by the abdominal walls, and thereby prevents the afflux and stasis of the fluids, the engorgement of the uterine walls and the dilatation of the cavity of this viscus; and it has the further advantage of obviating the tendency to cyncope, and of diminishing the after-pains. But, in order to obtain all these benefits, it should be large enough to compress the whole sub-umbilical regions equally."

Francis H. Ramsbotham, M. D., Principles and Practice of Obstetric Medicine and Surgery, says: "The principal object which the bandage serves is to brace the bowels, and give an artificial support in lieu of that which they have lost through the laxity of the abdominal muscles, and to prevent the faintness frequently attendant on the sudden removal of a certain degree of pressure." Prof. Churchill says: "I do not know that we can consider the binder *absolutely* necessary. * * * * * I am satisfied that it is very useful at first in maintaining a certain degree of contraction of the uterus, and giving support to the abdomen, and, afterwards in promoting a return to the natural condition of the uterine and abdominal parietes; for which reason I think it deserving of rather more attention than is usually paid to it, at least after the first day or two." D. Francis Condie strongly advocates the use of the appliance, and regards it as an additional safeguard against hemorrhage through its agency in stimulating uterine contractions.

Prof. Meigs, of Philiadelphia, during the latter years of his life, strongly conveys the impression to the mind of the young obstetrician that in every case of midwifery the application of the *post partum* binder is an unalterable law which it is his imperative duty to observe always. To Dr. William Corson, of Montgomery county, Penn., is imputed the distinction of having led off in the practice of the abandonment of the binder in women after parturition, in this country. In a practice of fifteen years preceding 1870, he claims to have dispensed with its use in fifteen hundred cases, and another physician of his state dispensed with it in one thousand cases. Dr. Hiram Corson, a brother of Dr. Wm. Corson in several hundred cases as also, some others mentioned in the same report with a large number of cases, and there have been no bad results from the practice in any single case, but, on the contrary, so great was the satisfaction expressed by the women that their warmest praise of the plan was e'icited. Dr. Hays, of the Hartfort County Pennsylvania Medical Society, says he is satisfied, prolapsus of the uterus has been produced by the mal-application of the binder, and mentions the case of a patient who had suffered from prolapsus after each of several previous confinements, in which she had worn bandages. In a subsequent case, by omitting the use of it, and lying in bed a week longer, she was completely cured. In the Transactions of his society of 1873, Dr. Hiram Corson give a well authenticated case, in which a physician in one of our large cities came daily to a lady for eighteen days, to lighten the bandage. Afterwards, on rising, she found herself with an aggravated case of prolapsus uteri. Nor was this all: so uncomfortable was she from the bandage, she trembled at the sight of her physician as he renewed his daily visits. I certainly fail to understand why the post partum binder should be resorted to as the sine qua non, whereby the relief of uterine hemorrhage is to be attained. I do readily admit the fact that pressure will stimulate uterine contraction, but I am of the opinion that this same pressure, which, momentarily applied, so promptly answered this end, will not continue to do so when prolonged beyond a certain point in time. Just as we are all aware that a momentary resistance to muscular action is seldom, if ever, followed by a neutralization of the muscular force, but if the effort be prolonged sufficiently, it will, beyond doubt, accomplish the end sought. And if this theory be correct, when applied in practice, the binder, in addition to the erroneous principle it illustrates, becomes an obstacle of magnitude, when the hurry of removal comes in that hour of fearful peril, when puerperal hemorrhage supervenes. Again, the claim has been made, that the prompt application of the means under consideration will effectually prevent that state of the anterior abdominal walls known as pendulous belly. Now the degree of enlargement which has been reached at the end of a full term gestation is very considerable, we admit, but it has not suddenly been made so great. This distension has been proceeding through the space of several months pari passu with the development of the fœtus and its appendages, so that, after the emission of the uterine contents, we do not find the muscular fibre of the abdominal wall rudely stretched—in many parts ruptured and torn, and other grave signs of *sudden distension;* but the parts have softened and drawn out gradually to the extent required, to accommodate the womb and its contents, and a full chance to shorten themselves is now given, and they will do it rapidly, unless the parturient rises prematurely, and even then I fail to see the causes which originate the pendulous belly which is so much spoken of and so greatly feared.

But the luxurious livers of this country, who are so negligent of all the laws of health, through ignorance, physiology and hygiene, or willful disregard of their teachings, require to be cared for during a few post-partum days. I should prefer the treatment by posture rather than risk the deleterious effects of the post-partum binder.

In those women who are in prime health and vigor of constitution, who are physiologically perfect, I assume the omission of the binder as being altogether safe. If not, why are we not startled by the tidings of constantly recurring instances of hemorrhagious uteri among the parturient females of Africa, or of the various uncivilized Indian tribes of North America. Many of these, we are authentically informed, are taken in labor on the march, their lying-in couch is the bosom of mother earth, their roof the hospitable broad canopy of the Heavens, whilst their accoucheur may be none other than Dame Nature, assisted in the necessary manipulations of the child by their own maternal hands; and yet, immediately they strap their offspring to their backs and proceed upon their weary tramp, unwrapped by a binder. Descending a step in the scale, we find examples of quadrupedal parturients, whose bellies are always pendant, that are never blessed with the luxury of a binder, and yet it would be difficult to find a class which is more exempt from trouble in the bringing forth of their offspring and the attendant phenomena. But we will meet with those who demand the application of the bandage in the face of all we may say or do to convince them of the uselessness of it; and if we fail to see it applied ourselves, it will be done by some one in our absence, for whose ignorance and folly popular opinion will hold us responsible. In such cases where it must be applied, I think we should see to the proper *construction* of it, as well as proper application of it, in order that serious harm may not result from its use. Instead of having an ordinary long towel, or some similarly shaped cloth, simply pinned tightly around the abdomen, as is generally done, have a binder constructed with a gusset so that it can be made to fit closely and uniformly over the lower part of the abdominal parietes, and thus be rendered secure from the danger of slipping upward, and gathering in a cord-shaped mass around the waist, creating such pressure as to insure a prolapsed uterus in the end; besides, let this binder be so applied as to exert simply a firm support, and not an undue amount of pressure. We are anxious to supply a *deficiency* in a natural condition, not anything *de novo.*

In summing up the disadvantages of the binder as a post-partum measure, Dr. Cairns, of Edinburgh, Scotland, enumerates the following, namely:

1. That their application entails unnecessary trouble upon the accoucheur. Dr. Cairns confesses that when he first entered upon practice, it cost him more trouble to apply the binder, in many cases, than to deliver either the child or placenta.

2. That their application exposes the patient unnecessarily, which, if several persons are present, may thereby shock her moral sensibilities. It may, moreover, expose her to currents of cold air, which, on her part, may lead to disastrous results.

3. Post-partum binders impede the circulation, slipping far above the region of the uterus, thus interfering with the veinous circulation, and thus tending to aggravate two diseases very common in pregnant women, viz: Varicose veins and hemorrhoid.

4. They are rarely of proper form. They should, properly, extend from the ensiform cartilage to a considerable way beyond the nates.

5. In cases of post-partum hemorrhage, the patient may die before the binders can be removed, in order to apply the proper remedies for its arrestment.

---

### PLASTER-PARIS IN THE TREATMENT OF FRACTURES.

BY E. H. TRICKLE, M. D., OF SYRACUSE, OHIO.

*Read before the Meigs County (Ohio) Medical Society.*

